# Resiliência Cardiopulmonar em Indivíduos Altamente Ativos: Análise de Testes de Esforço Cardiopulmonar Pré e Pós-Covid-19

**DOI:** 10.36660/abc.20250094

**Published:** 2025-10-28

**Authors:** Fabrício Braga, Gabriel Espinosa, Amanda Monteiro, Mauricio Milani, Juliana Paiva, Juliana Goulart Prata Oliveira Milani, Leandro Franzoni, Ricardo Stein, Gerson Cipriano, Jonas Lírio Gurgel, Ricardo Mourilhe-Rocha

**Affiliations:** 1 Laboratório de Performance Humana Rio de Janeiro RJ Brasil Laboratório de Performance Humana, Rio de Janeiro, RJ – Brasil; 2 Universidade do Estado do Rio de Janeiro Rio de Janeiro RJ Brasil Universidade do Estado do Rio de Janeiro, Rio de Janeiro, RJ – Brasil; 3 Universidade de Brasília (UnB) Programa de Pós-Graduação em Ciências Tecnologias da Saúde Brasília DF Brasil Programa de Pós-Graduação em Ciências e Tecnologias da Saúde, Universidade de Brasília (UnB), Brasília, DF – Brasil; 4 Hasselt University REVAL/BIOMED Hasselt Bélgica Hasselt University, REVAL/BIOMED, Hasselt – Bélgica; 5 Heart Centre Hasselt, Jessa Hospital Hasselt Bélgica Heart Centre Hasselt, Jessa Hospital, Hasselt – Bélgica; 6 Universidade Federal do Rio de Janeiro Rio de Janeiro RJ Brasil Universidade Federal do Rio de Janeiro, Rio de Janeiro, RJ – Brasil; 7 Universidade de Brasília Brasília DF Brasil Universidade de Brasília, Brasília, DF – Brasil; 8 Universidade Federal do Rio Grande do Sul Porto Alegre RS Brasil Universidade Federal do Rio Grande do Sul, Porto Alegre, RS – Brasil; 9 Universidade Federal Fluminense Niterói RJ Brasil Universidade Federal Fluminense, Niterói, RJ – Brasil; 10 Complexo Hospitalar Américas- Vitória e Samaritano Barra Rio de Janeiro RJ Brasil Complexo Hospitalar Américas- Vitória e Samaritano Barra, Rio de Janeiro, RJ – Brasil

**Keywords:** Aptidão Cardiorrespiratória, Exercício Físico, Teste de Esforço, Desempenho Atlético

## Abstract

**Fundamento:**

A pandemia de covid-19 afetou milhões de pessoas em todo o mundo, com impactos persistentes que se estendem além da fase aguda. Um desses efeitos é a condição conhecida como pós-covid (ou covid longa), caracterizada por sintomas como fadiga e intolerância ao exercício com duração superior a 60 dias. Embora o exercício físico regular esteja associado à redução do risco de desfechos graves, relatos de queda no desempenho atlético após a infecção — mesmo entre indivíduos altamente ativos (IAAs) — têm gerado preocupações quanto aos efeitos de longo prazo sobre a saúde física. O teste de esforço cardiopulmonar (TECP) é uma ferramenta valiosa para avaliar a intolerância ao exercício e investigar as consequências metabólicas e ventilatórias da covid-19.

**Objetivo:**

Avaliar o impacto da covid-19 na função cardiopulmonar de IAAs por meio da análise das respostas metabólicas e ventilatórias obtidas em TECP realizado antes e após a infecção.

**Métodos:**

Foram analisados retrospectivamente dados de TECP de IAAs de ambos os sexos. Os desfechos primários incluíram alterações no consumo máximo de oxigênio ( $\overset{\cdot }{\text{V}}$ O_2_pico) e na eficiência ventilatória (relação $\overset{\cdot }{\text{V}}$ E/ $\overset{\cdot }{\text{V}}$ CO_2_). O nível de significância estatística foi estabelecido em 5% (p < 0,05).

**Resultados:**

Foram incluídos 43 IAAs (72,1% do sexo masculino; 44 ± 10 anos). O intervalo mediano entre os testes foi de 479 dias, sendo o segundo TECP realizado em média 44 ± 27 dias após a infecção por covid-19. Observou-se uma redução média de 1,5 ml/kg/min no $\overset{\cdot }{\text{V}}$ O_2_pico (p = 0,017), correspondendo a uma diminuição de 3,84% nos valores previstos de $\overset{\cdot }{\text{V}}$ O_2_pico (p = 0,045). A relação $\overset{\cdot }{\text{V}}$ E/ $\overset{\cdot }{\text{V}}$ CO_2_ aumentou em média 1,2 (p = 0,017).

**Conclusão:**

Embora os IAAs não sejam imunes aos efeitos da covid-19, seu elevado nível basal de atividade física parece conferir uma considerável resiliência cardiopulmonar. As alterações observadas após a infecção foram mínimas, sugerindo que a manutenção da aptidão física pode oferecer benefícios protetores contra sequelas prolongadas da doença.

## Introdução

A pandemia de covid-19, iniciada em dezembro de 2019 em Wuhan, China, já afetou mais de 600 milhões de pessoas e resultou em mais de 6 milhões de mortes em todo o mundo ao longo de cinco ondas de infecção nos últimos 3 anos.^1^ Os sintomas da covid-19 são conhecidos por persistirem além da fase aguda, resultando em sequelas pós-agudas denominadas síndrome pós-covid (ou covid longa), caracterizada por manifestações clínicas com duração superior a 60 dias.^2 , 3^ Embora os mecanismos subjacentes ainda não estejam completamente elucidados, a fadiga e a intolerância ao exercício estão entre os sintomas mais frequentemente relatados.^4^

O teste de esforço cardiopulmonar (TECP) é considerado o padrão-ouro para avaliação da intolerância ao esforço e é recomendado na investigação de indivíduos com covid longa.^5 – 7^ Apesar dos efeitos protetores bem estabelecidos da prática regular de atividade física e da elevada aptidão cardiorrespiratória (ACR) contra formas graves da doença e suas sequelas, até mesmo atletas têm relatado redução no desempenho físico após infecção por covid-19.^8^ Estudos baseados em TECP descreveram diversas alterações nessa população, incluindo redução da ACR, início precoce do metabolismo anaeróbio e padrões ventilatórios anormais. No entanto, poucos estudos compararam dados de TECP realizados antes e após a infecção em indivíduos fisicamente ativos.

Dessa forma, o objetivo deste estudo foi comparar as respostas metabólicas e ventilatórias obtidas por meio de TECP realizado antes e depois da infecção por covid-19 em indivíduos altamente ativos (IAAs).^9^

**Figure:**
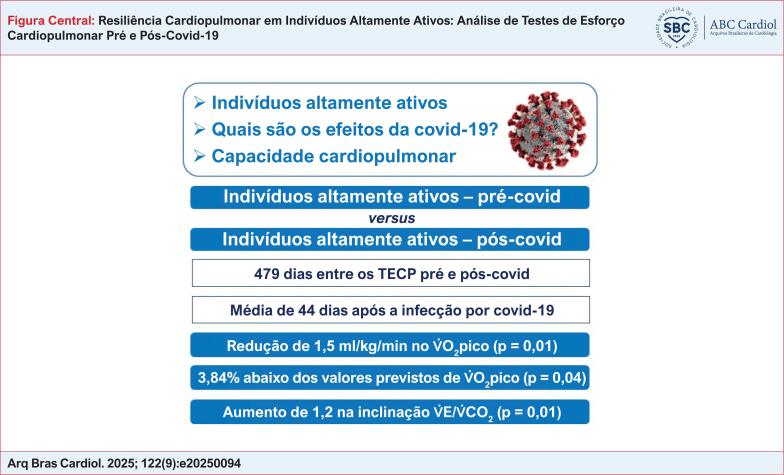


## Métodos

Este estudo transversal envolveu uma análise *ex post facto* de dados de TECP coletados entre maio de 2020 e setembro de 2022 em dois centros de cardiologia do esporte. Foram considerados elegíveis os indivíduos que atendiam a todos os seguintes critérios: (1) idade ≥ 18 anos; (2) nível 4 na Saltin-Grimby Physical Activity Level Scale (SGPALS), o que indica treinamento intenso e regular para esportes competitivos;^10^ (3) TECP realizado como parte da avaliação pós-covid-19 antes da retomada de treinos competitivos com alto volume ou alta intensidade; e (4) disponibilidade de um TECP pré-covid-19 realizado no mesmo ergômetro.

O estudo foi aprovado pelos comitês de ética das duas instituições participantes (CAAE 33729120.5.0000.5253 e 35706720.4.0000.8093). Todos os procedimentos foram conduzidos de acordo com a Declaração de Helsinque da Associação Médica Mundial, versão de 2013.

O TECP foi realizado em esteira ergométrica (Centurium 200) ou em cicloergômetro com freio eletromagnético (Schoberer Rad Meßtechnik [SRM], Alemanha), com análise de gases respiratórios em tempo real ( *breath-by-breath* ) utilizando o sistema Metalyzer 3B (Cortex). Nos testes realizados em cicloergômetro, a geometria da bicicleta foi ajustada conforme as preferências individuais, e os participantes foram orientados a utilizar vestuário adequado para ciclismo. O protocolo teve início com 2 minutos de repouso, seguidos por 5 minutos de aquecimento em carga constante (100 W para homens e 50 W para mulheres). Em seguida, foi aplicado um protocolo em rampa, com incrementos de carga de 25 W/min para homens e 15 W/min para mulheres, até a exaustão voluntária. Os participantes foram instruídos a manter uma cadência compatível com seus treinos habituais, sendo o teste encerrado quando a cadência caía abaixo de 70 rpm, mesmo com incentivo verbal. Nos testes realizados em esteira, utilizou-se um protocolo em rampa individualizado, com velocidade inicial entre 4 e 6 km/h, aumentada de forma linear conforme a idade e o nível de desempenho atlético autorreferido pelo participante. Para garantir sobrecarga progressiva ao longo do teste, foi incluído um aumento de inclinação de 0,5% a cada 2 minutos. Todos os procedimentos seguiram as recomendações nacionais de mitigação da transmissão viral.^11^

O protocolo e a metodologia para a análise de gases ventilatórios, conforme previamente descrito,^12 , 13^ incluíram espirometria pré-exercício para avaliação da capacidade vital forçada (CVF) e do volume expiratório forçado no primeiro segundo (VEF_1_). As variáveis analisadas no TECP incluíram consumo de oxigênio ( $\overset{\cdot }{\text{V}}$ O_2_), frequência cardíaca (FC), frequência respiratória (FR), volume corrente (VC) e ventilação ( $\overset{\cdot }{\text{V}}$ E) nos primeiros (VT_1_) e segundos (VT_2_) limiares ventilatórios, bem como no pico do exercício. A eficiência ventilatória foi avaliada por meio do equivalente ventilatório para o dióxido de carbono ( $\overset{\cdot }{\text{V}}$ E/ $\overset{\cdot }{\text{V}}$ CO_2_) nos momentos de VT1, VT_2_, pico e também pela inclinação da relação $\overset{\cdot }{\text{V}}$ E/ $\overset{\cdot }{\text{V}}$ CO_2_.

Um médico experiente determinou os limiares ventilatórios e os valores de pico. A inclinação $\overset{\cdot }{\text{V}}$ E/ $\overset{\cdot }{\text{V}}$ CO_2_ foi calculada até o VT_2_. Os valores de $\overset{\cdot }{\text{V}}$ O_2_pico e $\overset{\cdot }{\text{V}}$ E foram definidos como a maior média móvel de 30 segundos registrada durante o último minuto de esforço. Os valores previstos de $\overset{\cdot }{\text{V}}$ O_2_pico foram baseados em padrões de referência nacionais estabelecidos e utilizados para a classificação da aptidão cardiorrespiratória (ACR).^14^

A variação percentual (Δ%) no $\overset{\cdot }{\text{V}}$ O_2_pico e na relação $\overset{\cdot }{\text{V}}$ E/ $\overset{\cdot }{\text{V}}$ CO_2_ foi calculada como a diferença entre os valores pós-covid-19 e pré-covid-19, dividida pelo valor pré-COVID-19. Essas variações foram comparadas com as diferenças críticas ( *critical differences* , CD) propostas por Rose et al. (2018) — 13% para o $\overset{\cdot }{\text{V}}$ O_2_pico e 10% para a relação $\overset{\cdot }{\text{V}}$ E/ $\overset{\cdot }{\text{V}}$ CO_2_.^15^ Esse limiar foi utilizado para determinar se as alterações observadas poderiam ter relevância clínica e biológica.

### Análise estatística

Dado o caráter exploratório deste estudo, não foi realizado cálculo de tamanho amostral a priori. Em vez disso, adotou-se um delineamento abrangente, incluindo todos os indivíduos que atendiam aos critérios de elegibilidade. As variáveis contínuas foram expressas como média ± desvio padrão (DP) para dados com distribuição normal, ou como mediana e intervalo interquartílico (25° percentil; 75° percentil) para dados com distribuição não normal, conforme determinado pelo teste de Shapiro-Wilk. Para comparação de dados paramétricos, utilizou-se o teste *t* pareado; para dados não paramétricos, foi aplicado o teste de postos sinalizados de Wilcoxon. As diferenças medianas para variáveis não paramétricas foram estimadas pelo método de Hodges-Lehmann.

O nível de significância estatística foi estabelecido em p < 0,05 para todas as análises. Todos os procedimentos estatísticos foram realizados utilizando o software IBM SPSS Statistics for Windows, versão 29.0 (IBM Corp., Armonk, NY, EUA).

## Resultados

**Figura 1:**
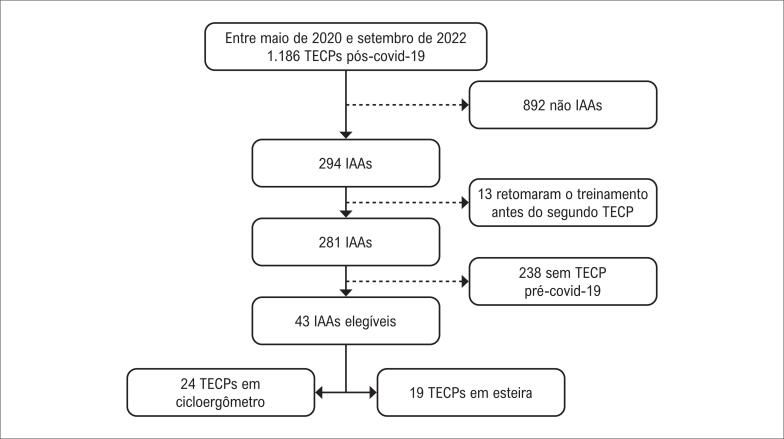
Fluxograma de inclusão dos participantes com base nos critérios de elegibilidade. IAA: indivíduo altamente ativo; TECP: teste de esforço cardiopulmonar.

A Figura 1 apresenta o fluxograma de inclusão dos participantes. Um total de 43 indivíduos altamente ativos (IAAs) atendeu aos critérios de inclusão e foi incluído na análise (72,1% do sexo masculino; 42 ± 10 anos). O tempo médio entre o diagnóstico de covid-19 e a realização do TECP pós-infecção foi de 44 ± 27 dias. O intervalo mediano entre os TECPs pré e pós-infecção foi de 479 dias (IIQ: 546 dias). O TECP foi realizado em cicloergômetro em 55,8% dos casos. Quase todos os participantes apresentaram quadro agudo leve, e nenhum necessitou de hospitalização.

A Tabela 1 resume as variáveis do TECP antes e após a infecção por covid-19. Não foram observadas alterações significativas nos parâmetros espirométricos (CVF e VEF_1_). No entanto, foram identificadas alterações discretas, porém estatisticamente significativas, em algumas variáveis do TECP. Tanto o $\overset{\cdot }{\text{V}}$ O_2_ no VT_2_ quanto o $\overset{\cdot }{\text{V}}$ O_2_pico apresentaram leves reduções. A variação percentual mediana no $\overset{\cdot }{\text{V}}$ O_2_pico foi inferior ao limite de −13% considerado clinicamente significativo (p ≤ 0,001). Apenas seis participantes (14%) apresentaram redução no $\overset{\cdot }{\text{V}}$ O_2_pico superior a esse limiar após a covid-19.

**Tabela 1:** Variáveis do teste de exercício cardiopulmonar antes e após a infecção por covid-19 em indivíduos altamente ativos

Variável	Antes da covid-19	Após a covid-19	Diferença pareada (IC 95%)	Valor p
**Espirometria** b				
CVF, L c	4,75 ± 1,00	4,78 ± 1,03	−0,07 (−0,26 a −0,12)	0,455
VEF_1_, L c	3,79 ± 0,74	3,86 ± 0,84	0,003 (−0,10 a 0,10)	0,956
**TECP**				
VO_2_pico, l/min c	3,52 ± 0,74	3,42 ± 0,71	−0,10 (−0,20 a 0,002)	0,055
VO_2_pico, ml/kg/min c	47,3 ± 7,1	45,8 ± 7,3	−1,5 (−2,79 a −0,29)	0,017
VO_2_pico, % do previsto d	123,0 (111,3–141,2)	118,4 (109,6–137,0)	−3,84 (−7,9 a −0,08)	0,045
FC pico, bpm d	174 (165–181)	173 (163–182)	−0,5 (−2,5 a 1,5)	0,647
$\overset{\cdot }{\text{V}}$ E pico, l/min c	138,4 ± 32,6	134,4 ± 29,8	−4,0 (−9,5 a 1,6)	0,159
VC pico, l c	2,66 ± 0,59	2,67 ± 0,62	0,01 (−0,07 a 0,08)	0,792
FR pico, respirações/min d	54,0 (46,5–58,0)	49,7 (45,0–57,0)	−1,5 (−3,5 a 0,35)	0,079
$\overset{\cdot }{\text{V}}$ E/ $\overset{\cdot }{\text{V}}$ CO_2_ pico d	32,6 (31,3–34,7)	34,2 (32,0–36,6)	1,2 (0,25 a 2,20)	0,017
Inclinação $\overset{\cdot }{\text{V}}$ E/ $\overset{\cdot }{\text{V}}$ CO_2_ c	30,7 ± 4,0	31,3 ± 4,0	0,63 (−0,30 a 1,56)	0,176
OUES c	3727 ± 868	3474 ± 1025	−253 (−508 a 1)	0,051
RER pico c	1,16 ± 0,07	1,15 ± 0,07	−0,01 (−0,03 a 0,01)	0,453
VO_2_ no VT_1_, l/min c	2,26 ± 0,54	2,21 ± 0,49	−0,06 (−0,19 a 0,07)	0,366
VO_2_ no VT_1_, ml/kg/min c	30,6 ± 6,2	29,3 ± 7,5	−1,34 (−3,54 a 0,86)	0,226
FC no VT_1_, bpm d	133 (119–144)	133 (119–144)	0,00 (−4,0 a 4,5)	0,913
$\overset{\cdot }{\text{V}}$ C no VT_1_, l/min d	58,8 (46,3–71,1)	61,2 (49,5–69,3)	0,7 (−4,3 a 5,8)	0,213
FR no VT_1_, respirações/min c	28,8 ± 6,9	28,1 ± 7,0	−0,7 (−2,0 a 0,7)	0,330
VC no VT_1_, l d	1,96 (1,70–2,55)	2,20 (1,70–2,63)	0,07 (−0,06 a 0,21)	0,817
$\overset{\cdot }{\text{V}}$ E/ $\overset{\cdot }{\text{V}}$ CO_2_ no VT1 c	29,3 ± 4,0	29,3 ± 3,4	0,01 (−0,94 a 0,96)	0,980
VO_2_ no VT_2_, l/min d	3,36 (2,76–3,59)	2,96 (2,67–3,50)	−0,11 (−0,25 a 0,02)	0,085
VO_2_ no VT_2_, ml/kg/minc	42,4 ± 6,0	40,6 ± 6,5	−1,71 (−3,32 a −0,10)	0,038
FC no VT_2_, bpm d	163 (155–173)	161 (150–169)	−0,5 (−3,5 a 2,5)	0,507
$\overset{\cdot }{\text{V}}$ E no VT_2_, l/min d	107,8 (82,8–119,4)	93,2 (83,8–113,3)	−3,7 (−8,5 a 1,43)	0,158
FR no VT_2_, respirações/min c	39,4 ± 8,1	37,6 ± 8,0	−1,8 (−3,50 a −0,06)	0,043
VC no VT_2_, l c	2,61 ± 0,60	2,62 ± 0,59	0,01 (−0,08 a 0,10)	0,833
$\overset{\cdot }{\text{V}}$ E/ $\overset{\cdot }{\text{V}}$ CO_2_ no VT_2_ c	30,3 ± 3,1	30,6 ± 3,2	0,31 (−0,42 a 1,04)	0,398

Os dados são apresentados como média ± DP ou mediana (IIQ), conforme apropriado.

a Diferença pareada = valor pós-covid-19 menos valor pré-covid-19; IC 95% fornecido.

b Dados espirométricos disponíveis para 30 indivíduos.

c Teste t pareado.

d Teste de postos sinalizados de Wilcoxon; método de Hodges-Lehmann aplicado para diferenças medianas. FR: frequência respiratória; TECP: teste de esforço cardiopulmonar; VEF1: volume expiratório forçado no primeiro segundo; CVF: capacidade vital forçada; FC: frequência cardíaca; OUES: inclinação da eficiência de captação de oxigênio; RER: razão de troca respiratória; $\overset{\cdot }{\text{V}}$ E: ventilação minuto; $\overset{\cdot }{\text{V}}$ E/ $\overset{\cdot }{\text{V}}$ CO_2_: equivalente ventilatório para dióxido de carbono; $\overset{\cdot }{\text{V}}$ O_2_: consumo de oxigênio; VT: limiar ventilatório; VC: volume corrente.

A relação $\overset{\cdot }{\text{V}}$ E/ $\overset{\cdot }{\text{V}}$ CO_2_ no pico aumentou significativamente após a infecção. No entanto, nenhum outro parâmetro de eficiência ventilatória apresentou alteração significativa. A variação percentual mediana na relação $\overset{\cdot }{\text{V}}$ E/ $\overset{\cdot }{\text{V}}$ CO_2_ no pico foi inferior ao limite de 10% estabelecido como CD (p < 0,001). Apenas dois participantes (4,7%) apresentaram aumento acima desse limite.

A FR no VT_2_ apresentou leve redução após a covid-19. Embora estatisticamente significativa, essa alteração não foi considerada clinicamente ou biologicamente relevante e não pode ser atribuída de forma definitiva aos efeitos pós-covid-19 ou à redução no volume de treinamento durante o período de recuperação.

## Discussão

Este estudo avaliou o impacto da infecção por covid-19 em IAAs e identificou reduções discretas no consumo de oxigênio tanto no pico do exercício quanto no VT_2_, além de um leve aumento na razão $\overset{\cdot }{\text{V}}$ E/ $\overset{\cdot }{\text{V}}$ CO_2_ no pico. Embora estatisticamente significativas, essas alterações não indicaram comprometimento cardiopulmonar relevante. Ademais, todas as variações observadas permaneceram abaixo dos limiares de CD previamente estabelecidos, o que sugere que é improvável que representem efeitos clinicamente significativos da covid-19 sobre a função cardiopulmonar. A Figura Central resume os principais achados do estudo.

Poucos estudos avaliaram os resultados do TECP em IAAs antes e após a infecção por covid-19. Śliż et al.^16^ relataram uma redução significativa no $\overset{\cdot }{\text{V}}$ O_2_pico, bem como no $\overset{\cdot }{\text{V}}$ O_2_ nos limiares ventilatórios VT_1_ e VT_2_ entre atletas de endurance. De forma semelhante aos nossos achados, esse estudo documentou uma redução de 5,9% no $\overset{\cdot }{\text{V}}$ O_2_pico — uma alteração que não pôde ser atribuída de forma conclusiva à covid-19. Nosso estudo oferece contribuições adicionais, ao incluir uma maior proporção de participantes do sexo feminino (27,9% vs 12,2%) e um intervalo mais curto entre a infecção e a realização do TECP pós-covid-19 (44 vs 155 dias), ampliando, assim, a compreensão atual sobre o possível impacto da covid-19 na função cardiopulmonar de IAAs.

Parpa e Michaelides^17^ avaliaram a ACR em jogadores de futebol profissional antes e após a infecção por covid-19 e relataram reduções significativas nos valores de $\overset{\cdot }{\text{V}}$ O_2_máx e no tempo de corrida em esteira após a infecção, mesmo após 60 dias de recuperação, sugerindo um impacto duradouro sobre a ACR. Em conjunto, esses resultados ressaltam os possíveis efeitos de longo prazo da covid-19 sobre o desempenho aeróbico em atletas. Vale destacar que nem o estudo de Parpa e Michaelides^17^ nem o de Śliż et al.^16^ incluíram avaliações da eficiência ventilatória.

No estudo de D’Isabel et al.^18^ realizado com bombeiros do Arizona, foram observadas reduções significativas no $\overset{\cdot }{\text{V}}$ O_2_pico e no $\overset{\cdot }{\text{V}}$ O_2_ no VT1 após a infecção. A diminuição média no VO_2_pico foi de 2,55 ml/kg/min, correspondendo a uma redução de 7,3% — a maior registrada até o momento. O $\overset{\cdot }{\text{V}}$ O_2_ no VT_1_ apresentou queda ainda mais acentuada, de 24,3%. Adicionalmente, observou-se piora na eficiência ventilatória, evidenciada pelo aumento da inclinação da relação $\overset{\cdot }{\text{V}}$ E/ $\overset{\cdot }{\text{V}}$ CO_2_ de 24,7 no período pré-infecção para 26,0 no pós-infecção. Diferentemente dos atletas de competição, os bombeiros do Arizona possuem rotinas de treinamento físico e exigências ocupacionais distintas, o que pode ter influenciado suas trajetórias de recuperação. Fatores demográficos e características regionais também podem ter contribuído para os desfechos observados. A natureza fisicamente extenuante do trabalho, somada à pressão adicional de responder a chamadas de emergência durante a pandemia, provavelmente gerou estresse elevado, o que pode ter prejudicado a recuperação cardiopulmonar e diferenciado essa experiência daquela vivida por populações atléticas.^19^

Por outro lado, Csulak et al.^20^ conduziram um estudo com nadadores de elite, avaliando o desempenho cardiopulmonar antes e após a infecção por covid-19. Os resultados não demonstraram alterações significativas nos principais parâmetros do TECP, incluindo $\overset{\cdot }{\text{V}}$ O_2_máx e $\overset{\cdot }{\text{V}}$ E/ $\overset{\cdot }{\text{V}}$ CO_2_, o que sugere que infecções leves por covid-19 tiveram impacto mínimo na função cardiopulmonar de atletas de alto rendimento.

As alterações observadas na capacidade aeróbica após a covid-19 entre IAAs podem não ser atribuídas exclusivamente ao vírus. Um estudo com atletas de endurance indicou que apenas duas semanas de destreinamento podem reduzir significativamente a função cardiopulmonar e a aptidão muscular. Isso sugere que a redução do treinamento durante o período de recuperação pode ter contribuído para o desempenho aeróbico diminuído observado em nosso estudo.^21^ Assim, é fundamental considerar o impacto da diminuição da atividade física ao interpretar alterações na função cardiopulmonar pós- covid-19.

As mudanças mínimas observadas na potência aeróbica máxima ( $\overset{\cdot }{\text{V}}$ O_2_pico) e a preservação da capacidade oxidativa submáxima ( $\overset{\cdot }{\text{V}}$ O_2_ no VT_1_) em nossa coorte podem ser atribuídas a diversos fatores. Pesquisas anteriores demonstraram que a atividade física pode atuar como fator protetor contra quadros graves, inclusive na covid-19.^12 , 22 , 23^ Reduções na ACR após a infecção têm sido associadas a formas graves da doença, idade avançada, sedentarismo e comorbidades.^24^ Por outro lado, o estilo de vida atlético, a ausência de comorbidades e a relativa juventude dos participantes de nosso estudo provavelmente contribuíram para a preservação da ACR. Além disso, a pandemia afetou o comportamento em relação ao exercício físico de maneiras diversas. Estima-se que o aumento da prática de atividade física durante esse período tenha variado de 9% a 33%.^25^ Por exemplo, os treinos virtuais de ciclismo apresentaram crescimento expressivo, impulsionados pelas medidas de confinamento, avanços tecnológicos e o apoio de grandes eventos como o Tour de France,^26^ acompanhados por um aumento de 170% nas vendas de equipamentos para treino indoor.^27 , 28^ Shaw et al.^29^ também relataram ausência de mudança no volume de treinamento entre ciclistas másters durante o confinamento. Esses achados sugerem que alguns indivíduos podem ter atingido um pico de condicionamento aeróbico antes da infecção, e que os valores observados no pós-covid-19 em nosso estudo podem refletir um retorno ao nível basal, em vez de um declínio real na função cardiopulmonar.

Este estudo apresenta diversas limitações relevantes. Primeiramente, a equivalência entre os parâmetros fisiológicos pré-covid-19 e os verdadeiros valores basais é incerta. Em segundo lugar, os comportamentos de treinamento no período pós- covid-19 e a gravidade da doença foram autorrelatados, o que introduz potencial viés de memória. Em terceiro lugar, a inclusão exclusiva de casos leves de covid-19 limita a generalização dos achados para indivíduos que vivenciaram quadros moderados ou graves. Em quarto lugar, a ausência de um grupo controle com menor nível de atividade física restringe a possibilidade de realizar inferências comparativas. Em quinto lugar, a coleta de dados ocorreu durante os estágios iniciais da pandemia, antes da disponibilidade de vacinas e do surgimento de variantes virais posteriores. Embora isso limite a aplicabilidade a outras fases da pandemia, também oferece um panorama único do impacto inicial da covid-19 em IAAs. Em sexto lugar, a população do estudo — caracterizada por idade média de 42 anos e $\overset{\cdot }{\text{V}}$ O_2_pico de 47 ml/kg/min — representa um grupo altamente ativo, mas que não corresponde ao nível de atletas de elite. Portanto, os achados podem não ser generalizáveis nem para atletas de alto rendimento, nem para indivíduos menos ativos. Em sétimo lugar, o uso de diferentes ergômetros (cicloergômetro vs esteira) e a ausência de análises estratificadas por sexo podem ter introduzido variabilidade adicional nas medidas fisiológicas. Por fim, a ausência de um grupo controle verdadeiro limita a capacidade de distinguir alterações pós-covid-19 de flutuações fisiológicas normais relacionadas a interrupções no treinamento ou a outros fatores não relacionados. Estudos futuros que incorporem grupos controle e um espectro mais amplo de níveis de aptidão física serão essenciais para fornecer evidências mais abrangentes e generalizáveis.

## Conclusão

Esta análise abrangente de IAAs comparou os resultados do TECP antes e após o início da pandemia de covid-19. Os resultados demonstraram uma notável resiliência cardiopulmonar do organismo em indivíduos com elevado nível de aptidão física. Embora tenham sido observadas alterações discretas na função cardiopulmonar, estas permaneceram abaixo dos limiares de CD, o que sugere uma capacidade preservada de resistir e se recuperar dos impactos fisiológicos da covid-19. Esses achados reforçam o potencial papel protetor de um estilo de vida ativo frente aos efeitos adversos de doenças infecciosas emergentes.

Disponibilidade de Dados

Os conteúdos subjacentes ao texto da pesquisa estão contidos no manuscrito.
