# Supplementary material for: Resiliência Cardiopulmonar em Indivíduos Altamente Ativos: Análise de Testes de Esforço Cardiopulmonar Pré e Pós-Covid-19
Source: Arq Bras Cardiol. 2025 Oct 28;122(9):e20250094. [Article in Portuguese] doi: 10.36660/abc.20250094 (PMC12671726; doi:10.36660/abc.20250094)
Supplement: Translation

# Translation: Cardiopulmonary Resilience in Highly Active Individuals: Pre–Post COVID-19 Cardiopulmonary Exercise Testing Analysis

**DOI:** 10.36660/abc.20250094i

**Authors:** Fabrício Braga, Gabriel Espinosa, Amanda Monteiro, Mauricio Milani, Juliana Paiva, Juliana Goulart Prata Oliveira Milani, Leandro Franzoni, Ricardo Stein, Gerson Cipriano, Jonas Lírio Gurgel, Ricardo Mourilhe-Rocha

**Affiliations:** 1 Rio de Janeiro RJ Brasil Laboratório de Performance Humana, Rio de Janeiro, RJ – Brasil; 2 Rio de Janeiro RJ Brasil Universidade do Estado do Rio de Janeiro, Rio de Janeiro, RJ – Brasil; 3 Brasília DF Brasil Programa de Pós-Graduação em Ciências e Tecnologias da Saúde, Universidade de Brasília (UnB), Brasília, DF – Brasil; 4 Hasselt Bélgica Hasselt University, REVAL/BIOMED, Hasselt – Bélgica; 5 Hasselt Bélgica Heart Centre Hasselt, Jessa Hospital, Hasselt – Bélgica; 6 Rio de Janeiro RJ Brasil Universidade Federal do Rio de Janeiro, Rio de Janeiro, RJ – Brasil; 7 Brasília DF Brasil Universidade de Brasília, Brasília, DF – Brasil; 8 Porto Alegre RS Brasil Universidade Federal do Rio Grande do Sul, Porto Alegre, RS – Brasil; 9 Niterói RJ Brasil Universidade Federal Fluminense, Niterói, RJ – Brasil; 10 Rio de Janeiro RJ Brasil Complexo Hospitalar Américas- Vitória e Samaritano Barra, Rio de Janeiro, RJ – Brasil

**Keywords:** Cardiorespiratory Fitness, Exercise, Exercise Test, Athletic Performance

## Abstract

**Background:**

The COVID-19 pandemic has affected millions globally, with persistent impacts extending beyond the acute phase. One such effect is post-COVID (long COVID), characterized by symptoms such as fatigue and exercise intolerance lasting more than 60 days. Although regular exercise is associated with reduced risk of severe outcomes, reports of decreased athletic performance after COVID-19 — even among highly active individuals (HAIs) — have raised concerns regarding the long-term effects on physical health. Cardiopulmonary exercise testing (CPET) is a valuable tool to assess exercise intolerance and to investigate the metabolic and ventilatory consequences of COVID-19.

**Objectives:**

To evaluate the impact of COVID-19 on cardiopulmonary function in HAIs by analyzing metabolic and ventilatory responses using CPET before and after infection.

**Methods:**

CPET data were retrospectively analyzed from HAIs of both sexes. Primary outcomes included changes in peak oxygen uptake ( $\overset{\cdot }{\text{V}}$ O_2_peak) and ventilatory efficiency ( $\overset{\cdot }{\text{V}}$ E/ $\overset{\cdot }{\text{V}}$ CO_2_slope). Statistical significance was set at 5% (p < 0.05).

**Results:**

A total of 43 HAIs (72.1% male; 44 ± 10 years) were included. The median interval between CPETs was 479 days, with testing performed a mean of 44 ± 27 days after COVID-19. $\overset{\cdot }{\text{V}}$ O_2_peak decreased by a mean of 1.5 mL/kg/min (p = 0.017), representing a 3.84% reduction in predicted $\overset{\cdot }{\text{V}}$ O_2_peak values (p = 0.045). $\overset{\cdot }{\text{V}}$ E/ $\overset{\cdot }{\text{V}}$ CO_2_slope increased by 1.2 (p = 0.017).

**Conclusion:**

Although HAIs are not immune to the effects of COVID-19, their high baseline physical activity levels appear to confer substantial cardiopulmonary resilience. Only minimal post-infection alterations were observed, which suggests that maintaining fitness may provide protective benefits against long-term sequelae of COVID-19.

## Introduction

The COVID-19 pandemic, which began in December 2019 in Wuhan, China, has affected more than 600 million people and resulted in over 6 million deaths worldwide across five waves of infection over the past 3 years.^1^COVID-19 symptoms are known to persist beyond the acute phase, resulting in post-acute sequelae referred to as long COVID syndrome, which is characterized by symptoms that last for more than 60 days.^2 , 3^Although the underlying mechanisms are not fully understood, fatigue and exercise intolerance are among the most frequently reported symptoms.^4^

Cardiopulmonary exercise testing (CPET) is the gold standard for evaluating exercise intolerance and is recommended for assessing individuals with long COVID.^5 – 7^Despite the well-established protective effects of regular physical activity and high cardiorespiratory fitness (CRF) against severe disease and its sequelae, even athletes have reported diminished physical performance following COVID-19 infection.^8^CPET-based studies have described several abnormalities in this population, including reduced CRF, early onset of anaerobic metabolism, and altered ventilatory patterns. However, few studies have compared pre- and post-infection CPET data in physically active individuals.

Therefore, the aim of this study was to compare metabolic and ventilatory responses using CPET performed before and after COVID-19 in highly active individuals (HAIs).^9^

## Methods

This cross-sectional study involved an ex post facto analysis of CPET data collected between May 2020 and September 2022 at two sports cardiology centers. Individuals were eligible for inclusion if they met all of the following criteria: (1) age ≥ 18 years; (2) level 4 on the Saltin-Grimby Physical Activity Level Scale (SGPALS), indicating intense regular training for competitive sports;^10^(3) CPET performed as part of a post–COVID-19 assessment prior to resuming high-volume or high-intensity competitive training; and (4) availability of a pre–COVID-19 CPET performed on the same ergometer.

The study was approved by the ethics committees of both participating institutions (CAAE 33729120.5.0000.5253 and 35706720.4.0000.8093). All procedures were conducted in accordance with the 2013 World Medical Association Declaration of Helsinki.

The CPET was performed using either a treadmill (Centurium 200) or an electromagnetically braked cycle ergometer (Schoberer Rad Meßtechnik [SRM], Germany) with breath-by-breath gas analysis (Metalyzer 3B, Cortex). For cycle ergometer tests, bike geometry was adjusted based on individual preferences, and participants were advised to wear appropriate cycling attire. The protocol began with 2 minutes of rest followed by a 5-minute warm-up at a constant workload (100 W for men, 50 W for women). This was followed by a ramp protocol with workload increments of 25 W/min for men and 15 W/min for women, continuing until volitional exhaustion. Participants were instructed to maintain a cadence consistent with their usual training, with the test terminating when cadence dropped below 70 rpm despite verbal encouragement. For treadmill tests, an individualized ramp protocol was used, with the initial speed set between 4 and 6 km/h and increased linearly based on the participant's age and reported athletic performance. A slight incline increase (0.5% every 2 minutes) was incorporated to ensure progressive loading throughout the test. All procedures adhered to national recommendations for viral transmission mitigation.^11^

The protocol and methodology for ventilatory gas analysis, as previously described,^12 , 13^included pre-exercise spirometry to assess forced vital capacity (FVC) and forced expiratory volume in 1 second (FEV^1^). CPET variables analyzed included oxygen consumption ( $\overset{\cdot }{\text{V}}$ O_2_), heart rate (HR), breathing frequency (BF), tidal volume (TV), and ventilation ( $\overset{\cdot }{\text{V}}$ E) at the first (VT^1^) and second (VT^2^) ventilatory thresholds, as well as at peak exercise. Ventilatory efficiency was assessed using the ventilatory equivalent for carbon dioxide ( $\overset{\cdot }{\text{V}}$ E/ $\overset{\cdot }{\text{V}}$ CO_2_) at VT^1^, VT^2^, peak, and as the $\overset{\cdot }{\text{V}}$ E/ $\overset{\cdot }{\text{V}}$ CO_2_slope.

An experienced physician determined ventilatory thresholds and peak values. The $\overset{\cdot }{\text{V}}$ E/ $\overset{\cdot }{\text{V}}$ CO_2_slope was calculated up to VT^2^. $\overset{\cdot }{\text{V}}$ O_2_peak and $\overset{\cdot }{\text{V}}$ E were defined as the highest 30-second moving average recorded during the final minute of exertion. Predicted $\overset{\cdot }{\text{V}}$ O_2_peak values were based on nationally established reference standards and were used to classify CRF.^14^

The percentage change (Δ%) in $\overset{\cdot }{\text{V}}$ O_2_peak and $\overset{\cdot }{\text{V}}$ E/ $\overset{\cdot }{\text{V}}$ CO_2_was calculated as the difference between post–COVID-19 and pre–COVID-19 values, divided by the pre–COVID-19 value. These changes were compared with the critical differences (CD) proposed by Rose et al. (2018) — 13% for $\overset{\cdot }{\text{V}}$ O_2_peak and 10% for $\overset{\cdot }{\text{V}}$ E/ $\overset{\cdot }{\text{V}}$ CO_2_.^15^This threshold was used to determine whether the observed changes were potentially clinically and biologically relevant.

### Statistical analysis

Since the exploratory nature of this study, a priori sample size calculation was not performed. Instead, a comprehensive design was employed, including all individuals who met the eligibility criteria. Continuous variables were reported as mean ± standard deviation (SD) for normally distributed data or as median with interquartile range (25th percentile; 75th percentile) for non-normally distributed data, as determined by the Shapiro-Wilk test. Paired *t* tests were used to compare parametric data, while the Wilcoxon signed rank test was used for nonparametric data. Median differences for nonparametric variables were estimated using the Hodges-Lehmann method.

Statistical significance was set at p < 0.05 for all analyses. All statistical procedures were performed using IBM SPSS Statistics for Windows, version 29.0 (IBM Corp., Armonk, N.Y., USA).

## Results

Figure 1 presents the patient inclusion flowchart. A total of 43 HAIs met the inclusion criteria and were included in the analysis (72.1% male; 42 ± 10 years). The mean time from COVID-19 diagnosis to post-infection CPET was 44 ± 27 days. The median interval between pre- and post-infection CPET was 479 days (IQR, 546 days). CPET was performed using a cycle ergometer in 55.8% of cases. Nearly all participants experienced mild acute illness, and none required hospitalization.

**Figure 2:** Patient inclusion flowchart based on the inclusion criteria. CPET: cardiopulmonary exercise testing; HAI: highly active individual.

Table 1 summarizes CPET variables before and after COVID-19. No significant changes were observed in spirometry parameters (FVC and FEV^1^). However, minor yet statistically significant changes were noted in selected CPET variables. Both $\overset{\cdot }{\text{V}}$ O_2_at VT^2^and $\overset{\cdot }{\text{V}}$ O_2_peak showed slight decreases. The median percentage change in $\overset{\cdot }{\text{V}}$ O_2_peak was below the −13% threshold for clinical significance (p ≤ 0.001). Only six participants (14%) exhibited a decrease in $\overset{\cdot }{\text{V}}$ O_2_peak exceeding this threshold after COVID-19.

**Table 1:** Cardiopulmonary exercise test variables before and after COVID-19 infection in highly active individuals

Variable	Before COVID-19	After COVID-19	Paired difference (95%CI)	p-value
**Spirometry** b				
FVC, L c	4.75 ± 1.00	4.78 ± 1.03	−0.07 (−0.26 a −0.12)	0.455
FEV_1_, L c	3.79 ± 0.74	3.86 ± 0.84	0.003 (−0.10 a 0.10)	0.956
**CPET**				
VO_2_peak, l/min c	3.52 ± 0.74	3.42 ± 0.71	−0.10 (−0.20 a 0.002)	0.055
VO_2_peak, ml/kg/min c	47.3 ± 7.1	45.8 ± 7.3	−1.5 (−2.79 a −0.29)	0.017
VO_2_peak, % do predicted d	123.0 (111.3–141.2)	118.4 (109.6–137.0)	−3.84 (−7.9 a −0.08)	0.045
Peak HR, bpm d	174 (165–181)	173 (163–182)	−0.5 (−2.5 a 1.5)	0.647
Peak $\overset{\cdot }{\text{V}}$ E, l/min c	138.4 ± 32.6	134.4 ± 29.8	−4.0 (−9.5 a 1.6)	0.159
Peak TV, l c	2.66 ± 0.59	2.67 ± 0.62	0.01 (−0.07 a 0.08)	0.792
Peak BF, breaths/min d	54.0 (46.5–58.0)	49.7 (45.0–57.0)	−1.5 (−3.5 a 0.35)	0.079
Peak $\overset{\cdot }{\text{V}}$ E/ $\overset{\cdot }{\text{V}}$ CO_2_ d	32.6 (31.3–34.7)	34.2 (32.0–36.6)	1.2 (0.25 a 2.20)	0.017
I $\overset{\cdot }{\text{V}}$ E/ $\overset{\cdot }{\text{V}}$ CO_2_slope c	30.7 ± 4.0	31.3 ± 4.0	0.63 (−0.30 a 1.56)	0.176
OUES c	3727 ± 868	3474 ± 1025	−253 (−508 a 1)	0.051
Peak RER c	1.16 ± 0.07	1.15 ± 0.07	−0.01 (−0.03 a 0.01)	0.453
VO_2_at VT_1_, l/min c	2.26 ± 0.54	2.21 ± 0.49	−0.06 (−0.19 a 0.07)	0.366
VO_2_at VT_1_, ml/kg/min c	30.6 ± 6.2	29.3 ± 7.5	−1.34 (−3.54 a 0.86)	0.226
FC at VT_1_, bpm d	133 (119–144)	133 (119–144)	0.00 (−4.0 a 4.5)	0.913
$\overset{\cdot }{\text{V}}$ C at VT1, l/min d	58.8 (46.3–71.1)	61.2 (49.5–69.3)	0.7 (−4.3 a 5.8)	0.213
FR at VT1, breaths/min c	28.8 ± 6.9	28.1 ± 7.0	−0.7 (−2.0 a 0.7)	0.330
VC at VT1, l d	1.96 (1.70–2.55)	2.20 (1.70–2.63)	0.07 (−0.06 a 0.21)	0.817
$\overset{\cdot }{\text{V}}$ E/ $\overset{\cdot }{\text{V}}$ CO_2_at VT1 c	29.3 ± 4.0	29.3 ± 3.4	0.01 (−0.94 a 0.96)	0.980
VO_2_at VT_2_, l/min d	3.36 (2.76–3.59)	2.96 (2.67–3.50)	−0.11 (−0.25 a 0.02)	0.085
VO_2_at VT_2_, ml/kg/min c	42.4 ± 6.0	40.6 ± 6.5	−1.71 (−3.32 a −0.10)	0.038
FC at VT_2_, bpm d	163 (155–173)	161 (150–169)	−0.5 (−3.5 a 2.5)	0.507
$\overset{\cdot }{\text{V}}$ E at VT_2_, l/min d	107.8 (82.8–119.4)	93.2 (83.8–113.3)	−3.7 (−8.5 a 1.43)	0.158
FR at VT_2_, breaths/min c	39.4 ± 8.1	37.6 ± 8.0	−1.8 (−3.50 a −0.06)	0.043
VC at VT_2_, l c	2.61 ± 0.60	2.62 ± 0.59	0.01 (−0.08 a 0.10)	0.833
$\overset{\cdot }{\text{V}}$ E/ $\overset{\cdot }{\text{V}}$ CO_2_at VT_2_ c	30.3 ± 3.1	30.6 ± 3.2	0.31 (−0.42 a 1.04)	0.398

Data are presented as mean ± SD or median (IQR), as appropriate.

a Paired difference = post–COVID-19 minus pre–COVID-19 value; 95%CI provided.

b Spirometry data available for 30 individuals.

c Paired t test used.

d Wilcoxon signed rank test used; Hodges-Lehmann method applied for median differences. BF: breathing frequency; CPET: cardiopulmonary exercise testing; FEV1: forced expiratory volume in 1 second; FVC: forced vital capacity; HR: heart rate; OUES: oxygen uptake efficiency slope; RER: respiratory exchange ratio; $\overset{\cdot }{\text{V}}$ E: ventilation; $\overset{\cdot }{\text{V}}$ E/ $\overset{\cdot }{\text{V}}$ CO2: ventilatory equivalent for carbon dioxide; VO_2_: oxygen uptake; VT: ventilatory threshold; TV: tidal volume.

The peak $\overset{\cdot }{\text{V}}$ E/ $\overset{\cdot }{\text{V}}$ CO_2_ratio increased significantly following COVID-19. However, no other ventilatory efficiency parameters demonstrated significant changes. The median percentage change in peak $\overset{\cdot }{\text{V}}$ E/ $\overset{\cdot }{\text{V}}$ CO_2_was below the 10% CD threshold (p < 0.001). Only two participants (4.7%) exhibited an increase exceeding the CD.

BF at VT^2^showed a slight post–COVID-19 reduction. Although statistically significant, these changes were not clinically or biologically meaningful and cannot be definitively attributed to post–COVID-19 effects or to reduced training volume during recovery.

## Discussion

This study evaluated the impact of COVID-19 infection on HAIs and identified minor reductions in oxygen consumption at both peak exercise and the VT^2^, along with a slight increase in the peak $\overset{\cdot }{\text{V}}$ E/ $\overset{\cdot }{\text{V}}$ CO_2_ratio. Although statistically significant, these changes did not indicate major cardiopulmonary impairment. Moreover, all observed variations remained below established CD thresholds, which suggests they are unlikely to reflect clinically meaningful effects of COVID-19 on cardiopulmonary function. Central Illustration summarizes the main findings of the study.

Few studies have assessed CPET results in HAIs before and after COVID-19 infection. Śliż et al.^16^reported a significant reduction in $\overset{\cdot }{\text{V}}$ O_2_peak as well as $\overset{\cdot }{\text{V}}$ O_2_at VT^1^and VT^2^among endurance athletes. Similar to our findings, their study documented a 5.9% decrease in $\overset{\cdot }{\text{V}}$ O_2_peak — a change not conclusively attributable to COVID-19. Our study contributes further insight, with a higher proportion of female participants (27.9% vs 12.2%) and a shorter interval between COVID-19 infection and post-infection CPET (44 vs 155 days), thereby enhancing the current understanding of the potential impact of COVID-19 on cardiopulmonary function in HAIs.

Parpa & Michaelides^17^evaluated CRF in professional soccer players before and after COVID-19 and reported significantly lower $\overset{\cdot }{\text{V}}$ O_2_max values and treadmill running time post-infection, even after 60 days of recovery, which suggests a lasting impact on CRF. Together, these results highlight the potential long-term effects of COVID-19 on aerobic performance in athletes. Notably, neither the study by Parpa & Michaelides^17^nor that by Śliż et al.^16^included assessments of ventilatory efficiency.

In the study by D’Isabel et al.^18^involving firefighters from Arizona, significant post–COVID-19 reductions were observed in both $\overset{\cdot }{\text{V}}$ O_2_peak and $\overset{\cdot }{\text{V}}$ O_2_at VT^1^. The mean decrease in $\overset{\cdot }{\text{V}}$ O_2_peak was 2.55 mL/kg/min, corresponding to a 7.3% reduction — the largest reported to date. $\overset{\cdot }{\text{V}}$ O_2_at VT^1^declined even more substantially, by 24.3%. Additionally, ventilatory efficiency worsened, as indicated by an increase in the $\overset{\cdot }{\text{V}}$ E/ $\overset{\cdot }{\text{V}}$ CO_2_slope from 24.7 pre-infection to 26.0 post-infection. Unlike competitive athletes, firefighters from Arizona have distinct physical training regimens and occupational demands, which may have influenced their recovery trajectories. Demographic factors and regional characteristics may also have contributed to the observed outcomes. The physically demanding nature of their work, combined with the added pressure of responding to emergency calls during the pandemic, likely introduced heightened stress, potentially impairing cardiopulmonary recovery and differentiating their experience from that of athletic populations.^19^

Conversely, Csulak et al.^20^conducted a study on elite swimmers, evaluating cardiopulmonary performance before and after COVID-19 infection. Their results showed no significant changes in key CPET parameters, including $\overset{\cdot }{\text{V}}$ O_2_max and $\overset{\cdot }{\text{V}}$ E/ $\overset{\cdot }{\text{V}}$ CO_2_, which suggests that mild COVID-19 infection had minimal impact on the cardiopulmonary function of high-level athletes.

The observed changes in aerobic capacity post-COVID-19 among HAIs may not solely be attributed to the virus. A study on endurance athletes indicated that just two weeks of detraining can significantly reduce cardiopulmonary function and muscular fitness. This suggests that the reduction in training during recovery from COVID-19 could contribute to the decreased aerobic performance observed in our study.^21^Therefore, it is crucial to consider the impact of reduced training activity when interpreting changes in cardiopulmonary function post-COVID-19.

The minimal changes observed in maximal aerobic power ( $\overset{\cdot }{\text{V}}$ O_2_peak) and the preservation of submaximal oxidative capacity ( $\overset{\cdot }{\text{V}}$ O_2_at VT^1^) in our cohort may be attributed to several factors. Prior research has demonstrated that physical activity can serve as a protective factor against severe illness, including COVID-19.^12 , 22 , 23^Reductions in CRF after COVID-19 have been associated with severe disease, older age, sedentary behavior, and comorbidities.^24^In contrast, the athletic lifestyle, absence of comorbidities, and relative youth of the individuals in our study likely contributed to the preservation of CRF. Furthermore, the COVID-19 pandemic affected exercise behavior in diverse ways. The increase in physical activity among individuals during this period is estimated to range from 9% to 33%.^25^For example, virtual cycling training saw marked growth, fueled by lockdown measures, technological advancements, and the endorsement of major events such as the Tour de France,^26^accompanied by a 170% increase in indoor training equipment sales.^27 , 28^Shaw et al.^29^also reported no change in training volume among master cyclists during confinement. These findings suggest that some individuals may have reached a peak in aerobic conditioning before infection, and the post–COVID-19 values observed in our study could represent a return to baseline rather than a true decline in cardiopulmonary function.

This study has several important limitations. First, the equivalence of pre–COVID-19 physiological parameters with true baseline values is uncertain. Second, post–COVID-19 training behaviors and illness severity were self-reported, which introduces potential recall bias. Third, the inclusion of only mild COVID-19 cases limits the generalizability of findings to individuals who experienced moderate or severe illness. Fourth, the lack of a less active control group restricts the ability to make comparative inferences. Fifth, data collection occurred during the early stages of the COVID-19 pandemic, prior to vaccine availability and the emergence of later viral variants. While this limits applicability to other phases of the pandemic, it also provides a unique snapshot of the initial impact of COVID-19 on HAIs. Sixth, the study population — characterized by a mean age of 42 years and a $\overset{\cdot }{\text{V}}$ O_2_peak of 47 mL/kg/min — reflects a group that is highly active but not at an elite athletic level. Therefore, the findings may not be generalizable to either elite athletes or less active individuals. Seventh, the use of different ergometers (cycle vs treadmill) and the absence of sex-specific analyses may have introduced additional variability in physiological measurements. Finally, the lack of a true control group limits the ability to distinguish post–COVID-19 changes from normal physiological fluctuations related to training interruptions or unrelated factors. Future studies incorporating control groups and a broader range of fitness levels will be essential to provide more comprehensive, generalizable insights.

## Conclusion

This comprehensive analysis of HAIs compared CPET results before and after the onset of the COVID-19 pandemic. The results demonstrated the body's remarkable cardiopulmonary resilience in the context of high physical fitness. Although minor changes in cardiopulmonary function were observed, these remained below CD thresholds, which suggests a preserved capacity to withstand and recover from the physiological impacts of COVID-19. These findings support the potential protective role of an active lifestyle against the adverse effects of emerging infectious diseases.

Data Availability

The underlying content of the research text is contained within the manuscript.
